# 

**Published:** 1870-05

**Authors:** 


### Books and Pamphlets Received.

Anatomy, Descriptive and Surgical. By Henry Gray, F. R. S., etc. With Drawings by H. V. Carter, M. D., also by Dr. Westmacott. Dissections by the Author, and Dr. Carter. An introduction on General Anatomy and Development by T. Holmes, M. A., Cantab. New American from the Fifth Enlarge[illegible] English Edition. With four hundred and sixty-two engravingson wood. Philadelphia: H. C. Lea. Received through T. Butler & Son.

The Indigestions; or Diseases of the Digestive Organs Functionally Treated. By Thomas R. Chambers, Honorary physician to H. R. H., the Prince of Wales; Lecturer on the Practice of Medicine at St. Mary's Hospital, etc. Third American Edition Revised. Philadelphia: H. C. Lea. Recived through T. Butler & Son.

Renal Diseases: a Chemical Guide to their Diagnosis and Treatment. By W. R. Basham, M. D., Fellow of the Royal College of Physicians, Senior Physician to Westminster Hospital, etc. With Illustrations. Philadelphia: H. C. Lea. Received through T. Butler & Son.

Spinal Irritation. By Wm. A. Hammond, M. D., Professor of Diseases of the Mind and Nervous System in Bellevue Hospital Medical College, etc. A paper read before the New York County Medical Society.

Medico-Legal Contributions. Case of the People against Elisha B. Fero. By Chas. H. Porter, M. D., Albany, N. Y.

Three cases of Imperforate Anus; with Remarks. By J. H. Pooley, M. D., Yonkers, N. Y.

Archives of Ophthalmology and Otology. By Professors J. H. Knapp, M. D., New York, and S. Moos, M. D., of Heidelburg. Vol. I. No. I. New York: Wm. Wood & Co.

The Transactions of the Medical Association of the State of Missouri. From its Reorganization, December 10th, 1867, to the Annual Meeting, April 27th, 1869.

Twenty-seventh Annual Report of the Managers of the State Lunatic Asylum, for the year 1869.

Annual Address before the Medical Society of the State of New York. By Prof. James P. White, M. D.

Valedictory Address before the Graduated Class of the National Medical College of Washington, D. C. By Prof. John Ordronaux, L. L. B., M. D.

Sixty-third Annual Circular of the Medical School of the University of Maryland. Session of 1870–1.

The Strange Defense of Dr. James H. Armsby. By Chas. A. Robertson, A. M., M. D., Albany, N. Y.

Correspondence concerning a Fatal case of Placenta Prævia. By Charles Buckingham, M. D. With an Appendix by D. Barnard.

